# Reversing resistance: different routes and common themes across pathogens

**DOI:** 10.1098/rspb.2017.1619

**Published:** 2017-09-27

**Authors:** Richard C. Allen, Jan Engelstädter, Sebastian Bonhoeffer, Bruce A. McDonald, Alex R. Hall

**Affiliations:** 1 Institute of Integrative Biology, ETH Zürich, CH-8092 Zurich, Switzerland; 2 School of Biological Sciences, The University of Queensland, Brisbane, Queensland 4072, Australia

**Keywords:** reversing resistance, biocides, antimicrobials, fungicides

## Abstract

Resistance spreads rapidly in pathogen or pest populations exposed to biocides, such as fungicides and antibiotics, and in many cases new biocides are in short supply. How can resistance be reversed in order to prolong the effectiveness of available treatments? Some key parameters affecting reversion of resistance are well known, such as the fitness cost of resistance. However, the population biological processes that actually cause resistance to persist or decline remain poorly characterized, and consequently our ability to manage reversion of resistance is limited. Where do susceptible genotypes that replace resistant lineages come from? What is the epidemiological scale of reversion? What information do we need to predict the mechanisms or likelihood of reversion? Here, we define some of the population biological processes that can drive reversion, using examples from a wide range of taxa and biocides. These processes differ primarily in the origin of revertant genotypes, but also in their sensitivity to factors such as coselection and compensatory evolution that can alter the rate of reversion, and the likelihood that resistance will re-emerge upon re-exposure to biocides. We therefore argue that discriminating among different types of reversion allows for better prediction of where resistance is most likely to persist.

## 1. Introduction

Resistance to a given biocide, such as an antibiotic or fungicide, can decline in frequency in populations no longer exposed to it (e.g. [1–4]), but often does not (e.g. [5–8]). Understanding what drives or prevents the decline of resistance would help predict the outcome of interventions like restricting antibiotic consumption, or multi-drug strategies aimed at minimizing resistance. Some evolutionary processes and genetic factors that alter the likelihood of reversion have been identified (e.g. coselection, costs of resistance, compensatory adaptation) and studied for specific resistance mechanisms in controlled experiments. In parallel, many studies have characterized changes in resistance, including associated genetic changes (e.g. [9]), in real-world pathogens and pests. Across systems however, it is unclear why reversion is more likely for some resistance mechanisms and ecological scenarios than others. Here, we identify some common themes of reversion among systems where resistance has been observed to decline. We draw on examples from various taxa including bacteria, protists, viruses, and fungi, because most of the key processes have been observed in some taxa or scenarios and not others. By doing so, we aim to alert researchers studying resistance in individual species or scenarios to mechanisms of reversion that may be important but so far only observed elsewhere.

**Figure 1.**
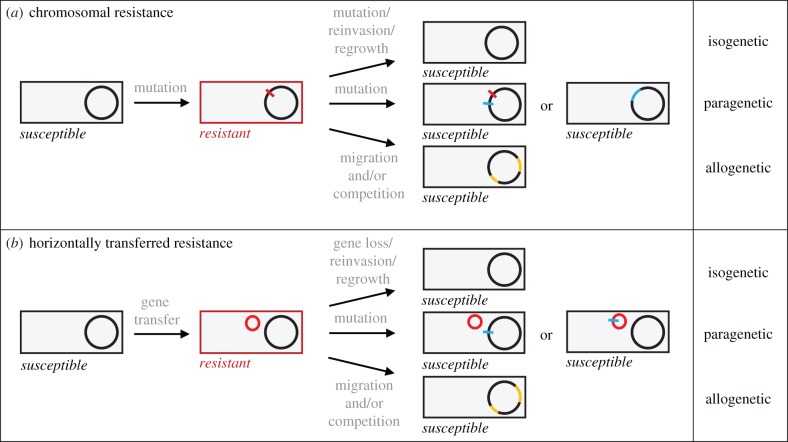
Alternative pathways to reversion. The grey rectangle represents a pathogen or pest, the black circle its genome. Resistance alleles are shown in red, other genetic changes affecting resistance in blue, and genetic variation at other loci in yellow. Resistance acquired by (*a*) mutation or (*b*) horizontal gene transfer may be reverted through isogenetic, paragenetic, or allogenetic processes. Some processes involve genetic changes to the prevailing genotype (isogenetic reversion via mutation or loss of horizontally transferred elements; paragenetic reversion), while others involve the prevailing genotype being replaced by a competing lineage of the same strain or species that is either the same as the ancestral genotype (isogenetic reversion via regrowth or reinvasion) or different (allogenetic reversion).

We first define the population biological processes that can reduce the average level of resistance in a population. We distinguish three types of reversion (figure 1): (i) resurgence of the ancestral, sensitive genotype that was prevalent before resistance evolution, (ii) acquisition of additional alleles by the resistant lineage that decrease resistance without restoring the ancestral genotype, (iii) replacement of resistant genotypes by less resistant genotypes of the same species or strain that are not derived from the ancestral population. We refer to these processes as isogenetic, paragenetic, and allogenetic reversion, respectively (figure 1), reflecting the different types of genetic variation involved. We give examples of each type and ask whether genetic or ecological information can explain which type is most likely in a given scenario, at what level it occurs (e.g. within-host versus among-host), and ultimately whether resistance is likely to persist or decline. A key parameter driving reversion is the fitness cost associated with resistance, which varies with genetic and environmental factors (e.g. [10,11]). This variation is important for predicting reversion and has been reviewed extensively elsewhere (e.g. [12]). However, the population biological processes by which costs of resistance translate to a net reduction in average resistance have received much less attention, and that is our focus here.

## 2. Isogenetic reversion by regrowth or reinvasion

Provided the ancestral sensitive population is not eliminated during treatment, it may increase from a reduced frequency after biocides are removed. The required coexistence of sensitive and resistant strains beyond treatment is most likely when biocide use is reduced after resistance begins to spread [13], which is the case in many clinical, agricultural, and veterinary settings. For example, WHO guidelines suggest reviewing treatments if the percentage of resistant *Neisseria gonorrhoeae* isolates obtained through routine surveillance exceeds 5% [14]. Even at the within-host scale, treated patients can harbour diverse populations in terms of antibiotic susceptibility [15]. Similarly, crop plants are often colonized by highly diverse populations of fungal pathogens (e.g. [16]), which can display significant variation in fungicide sensitivity even when sampled from the same field [17,18] or the same plant (e.g. [19]). In such scenarios, unlike mechanisms of reversion that first require the appearance of mutations or horizontal gene transfer, there is no waiting time for susceptible genotypes to arise and the likelihood that they are lost by genetic drift is relatively small as the initial revertant population is larger [20]. In line with this, Yang *et al.* [21] found reversion of transmitted drug resistance in HIV-1 was more likely in populations polymorphic at the locus involved in resistance.

The ancestral, sensitive genotype can survive treatment by remaining viable despite exposure to biocides, which subsequently permits reversion by regrowth from a reduced frequency, or because biocide concentrations are spatially heterogeneous, which permits reversion by reinvasion from untreated patches. In the former case, there are at least three processes allowing survival during exposure: (i) biocides inhibit growth of susceptible genotypes but are nonlethal, such as bacteriostatic or fungistatic compounds, (ii) susceptible individuals enter a phenotypic state permitting survival at otherwise lethal doses, such as bacterial biofilms or persisters, (iii) resistant individuals are present in sufficient numbers and resistance is encoded by an external detoxification mechanism, such as secreted deactivating enzymes or phospholipids [22] that reduce the effective concentration experienced by some susceptible individuals. Recent work suggests this increases survival of *Staphylococcus aureus* exposed to daptomycin in mice [23]. Such resistance mechanisms may be relatively likely to be associated with isogenetic reversion via regrowth.

Unlike regrowth, reinvasion can occur even when all susceptible individuals exposed to high biocide concentrations are killed. This requires sufficient spatial heterogeneity of biocide concentrations during treatment for the ancestral genotype to persist in some hosts or patches and sufficient pathogen migration for them to recolonize hosts or patches where resistant genotypes had spread during treatment. This could occur at the within-host scale for biocides with poor penetrance, such as the fungicide amphotericin B [24]. Resistance to amphotericin B in *Candida albicans* is very costly, increasing sensitivity to febrile temperatures, neutrophil attacks and other host-produced stressors *in vitro*, and reducing fitness in a mouse model [25]. Resistance rarely establishes in treated infections and susceptible *C. albicans* genotypes are isolated from treated hosts [24], suggesting reversion by reinvasion could be relevant here. Reinvasion is also possible at the among-host scale, and this may explain reversal of chloroquine resistance of *Plasmodium* in Malawi, which was found in most but not all infections at its peak and declined rapidly after chloroquine treatment was removed in 1993 [2,26]. In this case, the susceptible population was genetically diverse at other loci both before and after resistance/reversion [26] and likely persisted during the period of chloroquine use in asymptomatic, untreated infections. This suggests predicting reversion by reinvasion at the among-host scale requires not only monitoring the frequency of resistance in symptomatic infections but also nearby reservoirs or environmental compartments.

Reversion by reinvasion is likely to be common for plant pathogens because crops are typically planted in a patchwork where fungicide-treated fields can be adjacent to untreated fields. For example, Japanese populations of the rice blast pathogen *Pyricularia oryzae* became resistant to a fungicide affecting melanin biosynthesis 3 years after it was introduced [27]. Resistance was associated with a single nonsynonymous point mutation that occurred independently in three different *P. oryzae* lineages. After discontinuing the use of this fungicide, the frequency of resistant strains decreased from 72% to 25% in 1 year and was undetectable after 4 years. Much of the increase and decrease in resistance was due to spread of particular *P. oryzae* lineages including R-Sa4, R-Sa5, and R-Sa18 carrying resistance mutations [28]. These lineages showed higher fitness when the fungicide was present but were quickly replaced by other susceptible lineages, especially S-Sa5, after the fungicide was withdrawn [27]. Similarly, approximately 60% of strains of the sugar beet pathogen *Cercospora beticola* were resistant to triphenyltin hydroxide after approximately 14 years of continuous use in North Dakota and Minnesota [29], so most beet growers switched to fungicides with different modes of action. Ten years after switching fungicides, strains resistant to triphenyltin hydroxide were no longer detected. Soon thereafter, growers began to use triphenyltin hydroxide successfully. Both of these examples illustrate that biocides can be successfully reintroduced if fitness costs are high enough and pathogen migration among subpopulations is sufficiently high to enable susceptible alleles to reinvade from untreated areas while being sufficiently restricted to prevent movement of resistant alleles into new areas where the biocide is still effective. The plant pathologists working on *C. beticola* generate resistance maps annually to identify geographical hotspots of resistance to particular fungicides, and sugar beet growers in these hotspots are encouraged to use fungicides with a different mode of action in the following year to enable a reversion of resistance. This also suggests that alternating biocide applications may provide an effective long-term strategy to manage resistance.

## 3. Isogenetic reversion by mutation

If the ancestral, sensitive genotype that was prevalent before resistance evolution has been lost from the population it may be regained by reversal of the mutation(s) conferring resistance, as has been documented for a wide range of organisms including bacterial pathogens *in vitro* [30] and for HIV-1 in patients [21,31]. The likelihood of reversion via backward mutation depends on the supply rate of mutations restoring the ancestral genotype and their selective effects relative to alternative mutations at other loci [32], or pathogen migration. The likelihood of mutation back to the ancestral genotype will be greatly reduced when resistance results from multiple mutations, such as high-level resistance to fluoroquinolones in pathogenic bacteria [33], or when the genomic mutation rate is low.

Even when reversion requires a single backward mutation, there can be multiple possible beneficial mutations at other loci that have similar fitness benefits but do not reverse resistance. Experiments with pathogenic bacteria suggest acquisition of such compensatory mutations is more likely than reversion both *in vitro* [34] and *in vivo* [35]. In *Mycobacterium tuberculosis*, putative compensatory mutations have also been identified in clinical isolates [9]. Compensatory mutations are expected to have a fitness benefit contingent upon the presence of particular resistance alleles [36] or combinations of resistance alleles [37], meaning that reversion mutations are probably less beneficial, and therefore less likely to spread, after compensatory adaptation. In both *M. tuberculosis* and *Escherichia coli*, some mutations conferring resistance to other antibiotics also reduce the fitness costs of existing resistance alleles [38,39], indicating that switching from one antibiotic to another could simultaneously promote new resistance phenotypes and stabilize existing ones. Thus, isogenetic reversion via backward mutation is likely to be rare, as it can be precluded by mutations at other loci that are known to be segregating in real pathogen populations. Consistent with this, very few studies have reported this type of reversion outside the laboratory, with the notable exception of HIV-1 [21,31]. The reversibility of resistance in HIV-1 appears to vary among resistance alleles and may be explained by multiple factors, including high mutation rate [40], fitness costs associated with resistance [41], and that resistance can be encoded by a single amino acid change, meaning that a single mutation can restore the wild-type allele [42]. Because some of these factors also apply to other viruses, we can speculate that the supply of backward mutations may be relatively high for viruses compared to other types of pathogens.

## 4. Isogenetic reversion by loss of resistance genes

The rate at which revertant genotypes are generated is probably considerably higher for some resistance mechanisms on horizontally transferred elements than backward mutation of chromosomally encoded resistance. For example, plasmids can be lost through segregation at high rates [43] and this can rapidly reduce plasmid frequency in the absence of positive selection [44]. As with chromosomal mutations, the rate at which revertant genotypes displace resistant ones also depends on the cost of plasmid-encoded resistance [45] (which can be compensated by additional mutations [46]). The overall rate of decline will therefore depend on the balance of segregational loss, fitness costs, and conjugative transfer [47,48]. These parameters can change rapidly. For example, Smith [49] showed that despite the persistence of tetracycline-resistance genes in *E. coli* isolated from British pigs after prohibition of veterinary tetracycline use, the transmissibility of resistance among bacteria declined. Horizontally transmissible resistance alleles can also be maintained by post-segregational killing mechanisms such as toxin-antitoxin (TA) systems (unstable antitoxin; stable toxin) that make their loss deleterious to the host cell [50]. This could prevent isogenetic reversion and explain the prevalence of such plasmids in populations not consistently exposed to high antibiotic concentrations [51], including glycopeptide-resistant *Enterococcus faecium* [52].

When plasmid-encoded resistance genes are lost through segregation, they may be maintained in the local ‘resistome’ of the microbial community [53], and consequently may be rapidly regained by horizontal gene transfer if selecting antibiotics are reintroduced. This could occur if treatment promotes the spread of resistance in both the focal pathogen species and related lineages, and following the end of treatment resistance alleles are lost at different rates in different lineages. For example, Chung *et al.* [1] showed that resistance to ampicillin rapidly increased in *Haemophilus* isolates during treatment, but declined rapidly after treatment. This was correlated with changes in the frequency of a resistance element readily transferrable among related species including commensals [54]. Thus, although resistance declined in the focal pathogen species, the likelihood of re-evolving resistance upon re-exposure will depend on whether it had declined to a similar extent in other lineages that potentially constitute a reservoir of resistance genes.

Reversion by loss of resistance determinants from the same lineage that evolved resistance is not limited to plasmids, or even to horizontally transmitted elements. For example, for the human fungal pathogen *Candida albicans*, changes in fluconazole sensitivity were associated with aneuploidy caused by an isochromosome, an altered chromosome structure formed by fusing two left arms of chromosome 5, which carries the *ERG11* gene encoding the azole target protein. The isochromosome emerged in patients treated with fluconazole and then disappeared after treatment stopped [55].

## 5. Paragenetic reversion by mutation

Some genetic changes that diminish fitness costs can reduce resistance without reversing the original resistance allele. This appears to explain the reversal of chloroquine resistance in French Guiana, linked to additional mutations in the *pfcrt* gene involved in resistance [56]. That resistance in French Guiana reverted by this mechanism rather than reinvasion, as in Malawi, could reflect several differences in malaria disease dynamics and resistance evolution. The most important is probably that resistance in French Guiana, where transmission rates are low, appeared to fix during the period of chloroquine use [56]. Thus, following elimination of the ancestral genotype and without an influx of susceptible genotypes via transmission from other regions, reversion was only possible by genetic changes in the same lineage that evolved resistance. A similar process is thought to be happening for the CYP51 protein in plant-pathogenic fungi. Widespread use of azole fungicides over several decades in European wheat fields led to elimination of the ancestral wild-type CYP51 allele in *Zymoseptoria tritici*, which was replaced by a highly diverse array of CYP51 alleles that vary in their sensitivity to different members of the azole fungicide family [57], with evolved alleles conferring greater resistance to some azoles and greater sensitivity to other azoles [58]. Here, despite genetic variation at loci that determine resistance, reversion to the wild-type allele is virtually impossible.

In general, the mutational target size for paragenetic reversion is likely greater than that for isogenetic reversion. For example, *Salmonella enterica* Typhimurium and *S. aureus* can both recover the cost of mupirocin resistance by alternative mutations in *ileS*, the gene involved in resistance. These mutations increase fitness in the absence of antibiotics but reduce resistance [59,60], in some cases fully restoring susceptibility. Paragenetic reversion is also distinct from isogenetic reversion because the derived, reverted genotype is different from the pre-resistance-evolution ancestor. This is important because it could potentially alter the propensity to re-evolve resistance. For example, restrictions on antibiotic usage in Iceland were followed by an increase in the frequency of *Streptococcus pneumoniae* clones that retained resistance genes but had deactivated them, losing some of their resistance phenotypes [61]. Among the reverted clones, some had deleted large regions of resistance genes [61]. This should reduce the likelihood of them re-evolving resistance by mutation. Here, other clones including the original multi-resistant genotype also persisted, suggesting that both the mechanism of reversion and its extent in the pathogen population are important for predicting the likelihood that resistance re-emerges upon re-treatment. Deactivation of resistance by partially or completely deleting resistance genes has also been observed on resistance plasmids in *E. coli* [62,63]. This type of reversion is more likely for dedicated resistance genes that can be deactivated at little cost. This is because when resistance results from mutations in essential enzymes, such as RNA polymerase or the ribosome, deleting or deactivating these genes is lethal to bacteria.

## 6. Paragenetic reversion by modulating gene expression

Resistance can also be reduced without restoring the ancestral sequence by genetic changes that do not disrupt resistance genes themselves but alter their expression level. A recent study showed the importance of this process for ‘vancomycin-variable’ genotypes of *E. faecium*: an insertion sequence upstream of the *vanHAX* operon effectively silences the VanA resistance phenotype [64]. Critically, the resultant genotype is phenotypically sensitive but still possesses resistance genes, and the unstable nature of the insertion means that excision events are common. Consequently, resistant genotypes arise frequently via excision of the insertion sequence and spread rapidly upon treatment with vancomycin [64]. Thus, this mechanism of paragenetic reversion is associated with a relatively high propensity to re-evolve resistance upon re-exposure to selecting biocides. Silencing mutations that restore susceptibility but leave the bacteria capable of rapidly re-acquiring resistance have also been detected in plasmid-carrying *E. coli* [63].

Altered expression of resistance genes could also occur by reshuffling of integrons. Integrons are ‘gene capture devices’ found in many bacteria that can mediate the acquisition and expression of novel genes [65]. Their role in antibiotic resistance evolution is well established [66]: in many Gram-negative bacteria, antibiotic resistance genes have been acquired horizontally from other bacteria, incorporated into an integron and then selectively spread during treatment. However, integrons could also play an important but hitherto unexplored role during reversion once treatment is ceased. This is because the integrase, a recombinase that forms the core of an integron, can not only incorporate new genetic material but also excise and reshuffle the gene cassettes making up the integron. Consider a resistance gene cassette located close to the cassette promoter (*P*_c_, usually situated inside the integrase gene). Such a resistance gene will be highly expressed and thus provide strong resistance, but may also impose fitness costs [67]. In the absence of drug pressure, integrase-mediated reversion can occur in two ways: the cassette may be excised and lost, or other cassettes (either excised from further downstream within the integron or imported by horizontal gene transfer) may be integrated in the first position of the integron, so that the focal resistance cassette is moved downstream. Because genes farther from the cassette promoter (*P*_c_) are less highly expressed [68,69], continued reshuffling could reduce expression and associated fitness costs. Importantly, reversion by integron reshuffling need not be driven by selection to reduce fitness costs but could also be a by-product of selection for high expression of other cassettes, such as those encoding resistance to other drugs. Thus, reversion of integron-mediated resistance might occur rapidly even in the absence of fitness costs, provided other stressors with corresponding resistance gene cassettes are present. An important finding in this regard is that in class 1 integrons, integrase expression is regulated by the SOS response [70], which in turn is triggered by several antibiotics. Recent work indicates that cassette excision is more common than excision followed by re-insertion into the first position [69], which has important consequences for the capacity to re-evolve resistance upon re-exposure to selecting antibiotics: if the cassette is still present, increased expression may be regained by further reshuffling but if the cassette is lost it needs to be regained by horizontal gene transfer. In conclusion, integrons are expected to effect both rapid reversion and re-evolution of drug resistance, but we are only beginning to understand the population biological dynamics of these processes.

## 7. Allogenetic reversion within hosts

Under both isogenetic and paragenetic reversion the derived population of pathogens or pests is descended directly from the ancestral genotype that was present prior to resistance evolution. However, it is also important to account for interactions among different lineages of the same species or strain, because in many pathogens multiple genotypes are circulating within communities of hosts or within individual hosts [6,61,71]. Closely related strains and species can have characteristic differences in resistance phenotypes, such as penicillin susceptibility in *Streptococcus pyogenes* compared to related species [72], or the relatively high antibiotic resistance of *E. coli* from clonal complex 87 [73]. This means a lineage that has evolved resistance can potentially be outcompeted within a host by susceptible lineages of the same strain or species, such as those that superinfect the same host after biocide concentrations decline to a level conferring a competitive advantage to susceptibles. Reversion at the within-host scale appears to be common for antibiotic-resistant bacteria, as demonstrated by a recent meta-analysis [74]. Note that allogenetic reversion is different from clearance in that clearance is defined by a change in absolute pathogen or pest population size, whereas reversion is defined by a decline in average resistance in the population. Nevertheless, if a pathogenic, resistant lineage is replaced by a nonpathogenic, sensitive lineage, we could consider this to be simultaneous reversion and clearance.

**Figure 2.**
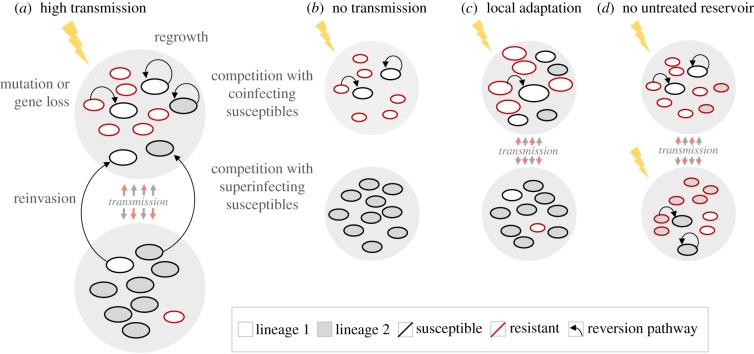
Within-host reversion is influenced by transmission and local adaptation. Two susceptible pathogen lineages (shaded and unshaded ovals; see legend) are unevenly distributed across two patches or hosts (large grey circles). Lightning indicates past treatment with a biocide that is no longer present, but favours costly resistant genotypes (red outline) that can emerge on either lineage. Size of the ovals represents expected fitness in the present host or patch; black arrows show possible pathways to reversion. (*a*) High transmission and costly resistance allow reversion by any process described above. (*b*) Lower transmission reduces the influx of susceptibles from other hosts/patches and the likelihood of coinfection, preventing both allogenetic reversion and isogenetic reversion by reinvasion. (*c*) Resistant genotypes are also locally adapted to their present host or patch, diminishing the competitive advantage of susceptibles and preventing isogenetic reversion by regrowth or reinvasion and allogenetic reversion resulting from outcompetition by coinfecting or superinfecting susceptibles; here the most likely reversion pathway is by mutation or gene loss (large white oval with black outline). Compensatory adaptation (not shown) will also reduce the advantage of susceptibles and the likelihood of reversion. (*d*) Biocide treatment across all hosts or patches reduces the supply of susceptible genotypes, making reversion through pathways requiring migration/gene flow less likely.

As with other types of reversion, allogenetic reversion is most likely when resistant pathogens incur large fitness costs. However, allogenetic reversion is also highly sensitive to the degree of local adaptation in the resistant population (figure 2): even if resistance itself is costly, resistant genotypes may be fitter than immigrating susceptibles if they also carry beneficial mutations affecting other traits relevant in the current environment. This could result, for example, from pathogens adapting to host factors or interactions with other microorganisms during chronic infection, as characterized extensively in *Pseudomonas aeruginosa* infections of cystic fibrosis patients [75]. Because allogenetic reversion at the within-host scale is less likely when transmission is too low for multiple genotypes to be present in the same host (figure 2), we can speculate that local adaptation is most likely to prevent reversion in either chronic infections or highly spatially structured populations, as opposed to scenarios with high gene flow among heterogeneously treated patches or hosts, such as in agricultural settings.

## 8. Allogenetic reversion at the among-host scale

Allogenetic reversion within hosts or patches relies on a supply of susceptibles and their competitive advantage over resistant genotypes in the same host or patch. However, allogenetic reversion can also be driven by resistant and susceptible genotypes colonizing new hosts or patches at different rates. Assuming a supply of uninfected or recovered hosts, pathogen genotypes with higher transmission rates will colonize vacant hosts or patches more frequently and consequently increase their frequency across the pathogen metapopulation (across all hosts or patches). Consistent with the idea that variable transmission rates could influence reversion, resistance to some antibiotics in *M. tuberculosis* can reduce transmission rate per infected host [76]. Resistance has also been associated with altered transmission in *Plasmodium* spp. [77]. In general, we can predict this type of reversion to be most likely for pathogens that are rapidly or widely transmitted relative to the duration or scale of treatment, providing a supply of susceptibles across different patches or hosts. This is particularly relevant for fungicides used against plant pathogens, where spatially heterogeneous biocide coverage is common.

## 9. Coselection

All of the above reversion mechanisms depend on the selective effects of resistance alleles, which can be modified by coselection: if resistance to a biocide is positively or negatively correlated with one or more other traits, selection on those traits can alter the frequency of biocide resistance [78]. Positive coselection of resistance mechanisms to multiple biocides (cross-resistance) is common and can result from a single mechanism conferring resistance to multiple biocides [79], or from two or more resistance genes occurring on the same genetic element, such as a multi-resistance plasmid or integron [5,80]. Coselection has been implicated in the lack of reversion of some types of antibiotic resistance in the absence of selecting drugs, because coselecting compounds were still in use [6,8]. On the other hand, recent *in vitro* work shows that resistance to some antibiotics increases bacterial susceptibility to other antibiotics (collateral sensitivity) [81,82], so that treatment with one may select for reversion of resistance to the other [10]. The relevance of reversion driven by collateral sensitivity remains unclear for bacterial pathogens outside the laboratory, but evidence from other pathogens suggests it can contribute to the efficacy of combination therapy. In HIV-1, the common clinical mutation M184 V of the reverse transcriptase (RT) enzyme confers resistance to 3TC (lamivudine) but suppresses the effect of AZT (zidovudine) resistance mechanisms in the same enzyme [83]. *In vitro*, AZT therapy alone or in combination with 3TC leads to reversion of the M184 V mutation [84], and similar reversion caused by sensitivity to PMPA (tenofovir) has been shown *in vivo* using macaques [85]. AZT and 3TC have been used together in fixed-dose combinations since 1997 and this is still considered an essential formulation [86].

In agriculture, collateral sensitivity is called negative cross-resistance and was exploited using mixture strategies that ultimately failed. Benzimidazoles inhibit ß-tubulin polymerization and prevent cell division in many species of fungi. Mutations in the ß-tubulin gene at codons 198 and 200 confer resistance to benzimidazoles. Two other fungicide classes, N-phenylcarbamates and benzamides, also inhibit ß-tubulin polymerization. Fungal strains sensitive to benzimidazoles are resistant to N-phenylcarbamates, while strains that are resistant to benzimidazoles are sensitive to N-phenylcarbamates. This allowed mixtures of these two fungicide classes to successfully control *Botrytis cinerea* in European vineyards from 1987 until 1994, when the F200Y mutation appeared which conferred resistance to both fungicide classes [87]. For N-phenylcarbamates and benzamides, negative cross-resistance depends on specific mutations present at codon 198 [88]. Wild-type isolates that are sensitive to benzimidazoles are insensitive to N-phenylcarbamates and benzamides. Isolates carrying the alanine E198A mutation lose sensitivity to benzimidazoles but become sensitive to N-phenylcarbamates and benzamides. However, another allele at this locus, E198 K, resulted in resistance to all three fungicide classes [89–91]. In such cases, alternative ways of deploying biocides, such as 2-way or 3-way alternations that exploit negative cross-resistance (collateral sensitivity) to drive recurrent rounds of reversion of single-resistance, may be more effective than using mixtures that consistently select for multi-resistance. However, pathogens may eventually overcome both types of treatment if generalized resistance mechanisms can evolve.

Coselection can also be driven by resistance being associated with traits other than resistance to additional biocides. For example, vaccines targeting resistant pneumococci led to a reduction in the prevalence of the strains included in the vaccine, which also had high resistance, indicating that an association between vaccine susceptibility and drug resistance can promote reversion [92]. Recent work suggests linkage with alleles affecting other aspects of pathogen life history, such as carriage duration, can also drive coselection during temporally variable exposure to antibiotics [93]. In general, the potential for coselection to influence reversion will depend on the extent to which resistance is correlated with other traits. In the simplest scenario, where resistance to a single biocide results from a single resistance gene that does not affect other traits and is not in linkage disequilibrium with other types of genetic variation under selection, coselection is unlikely to influence reversion. Alternatively, if resistance pleiotropically influences other traits (e.g. [94]) or is linked to other resistance genes [5], there is greater potential for coselection. For example, resistance to azole fungicides was pleiotropic with higher melanization and slower growth rates in *Z. tritici* [95] and with slower growth rates in *Rhynchosporium commune* [19]. Coselection driven by associations with other traits is particularly relevant for allogenetic reversion, where by definition resistant and sensitive genotypes differ at other loci beyond those encoding resistance. Moreover, when coselection is caused by genetic associations rather than pleiotropy it is expected to be more important in pathogens with low than with high rates of recombination or genetic exchange.

## 10. Conclusion

Why does discriminating among different types of reversion matter?

First, the risk of resistance re-emerging upon re-introduction of a biocide depends on the mechanism of reversion. For example, paragenetic reversion via unstable genetic changes, as for vancomycin variable enterococci [64], allows resistance to be rapidly regained upon re-exposure to antibiotics. The opposite effect is likely for paragenetic reversion by large deletions in resistance-determining regions, as observed in *S. pneumoniae* [61]. Therefore identifying the genetic basis of reversion, and ideally testing the stability of revertant phenotypes by culturing them *in vitro* or in animal models, permits predictions about the re-emergence of resistance.

Second, identifying likely mechanisms of reversion can inform experiments and surveillance. For example, experiments with homogeneous, resistant populations and no immigration are a poor model for scenarios where reversion is most likely via regrowth or reinvasion. This may explain the rarity of reversion in pure-culture evolution experiments compared to natural populations of bacterial pathogens [96]. Predicting reversion in such pathogens could be facilitated by measuring resistance for multiple clones from the same individual host or population (estimating sample diversity of resistance as well as the mean). In other scenarios, such as when resistance is reversed by loss of horizontally transferable elements that might persist in related members of the local microbial community, as observed for *Haemophilus* spp. [1,54], monitoring resistance genes beyond the focal pathogen could improve predictions. Such information is increasingly available through metagenomic/non-culture-based approaches to surveillance [97]. Finally, when asymptomatic infections are common and transmission is high enough to permit reinvasion, as for chloroquine resistance in Malawi [26], monitoring resistance beyond symptomatic, treated infections may help predict the potential for reversion by reinvasion.

Third, some reversion mechanisms, particularly coselection, can potentially be exploited to minimize the spread of resistance during treatment. These effects rely on successive rounds of reversion driven by negative cross-resistance/collateral sensitivity [10]. Negative cross-resistance has already been exploited with limited success against fungal pathogens [90]. For bacterial pathogens, results from resistant mutants isolated *in vitro* are promising [81,82], but implementing such strategies will require that resistance mechanisms in natural and clinical populations also display negative cross-resistance/collateral sensitivity. Ideally, this would be determined by measuring resistance profiles of isogenic strains with and without the resistance mechanisms circulating in pathogen populations, but the potential for managing resistance via coselection can also be inferred indirectly by measuring resistance profiles of clinical isolates, information that is already widely collected [15], and testing for non-random associations between resistance phenotypes.

## Data accessibility

This article has no additional data.
